# Impact of sex hormone-binding globulin on the human phenome

**DOI:** 10.1093/hmg/ddz269

**Published:** 2020-01-10

**Authors:** Ryan Arathimos, Louise A C Millard, Joshua A Bell, Caroline L Relton, Matthew Suderman

**Affiliations:** 1 Department of Population Health Sciences, Bristol Medical School, University of Bristol, Bristol, UK; 2 Medical Research Council Integrative Epidemiology Unit, University of Bristol, Bristol, UK; 3 Social Genetic and Developmental Psychiatry Centre, Institute of Psychiatry, Psychology & Neuroscience, King’s College London, London, UK; 4 NIHR Biomedical Research Centre for Mental Health, South London and Maudsley NHS Trust, London, UK; 5 Intelligent Systems Laboratory, University of Bristol, Bristol, UK

## Abstract

Background: Sex hormone-binding globulin (SHBG) is a circulating glycoprotein and a regulator of sex hormone levels, which has been shown to influence various traits and diseases. The molecular nature of SHBG makes it a feasible target for preventative or therapeutic interventions. A systematic study of its effects across the human phenome may uncover novel associations. Methods: We used a Mendelian randomization phenome-wide association study (MR-pheWAS) approach to systematically appraise the potential functions of SHBG while reducing potential biases such as confounding and reverse causation common to the literature. We searched for potential causal effects of SHBG in UK Biobank (*N* = 334 977) and followed-up our top findings using two-sample MR analyses to evaluate whether estimates may be biased due to horizontal pleiotropy. Results: Results of the MR-pheWAS across over 21 000 outcome phenotypes identified 12 phenotypes associated with genetically elevated SHBG after Bonferroni correction for multiple testing. Follow-up analysis using two-sample MR indicated the associations of increased natural log SHBG with higher impedance of the arms and whole body, lower pulse rate, lower bone density, higher odds of hip replacement, lower odds of high cholesterol or cholesterol medication use and higher odds of gallbladder removal. Conclusions: Our systematic MR-pheWAS of SHBG, which was comprehensive to the range of phenotypes available in UK Biobank, suggested that higher circulating SHBG affects the body impedance, bone density and cholesterol levels, among others. These phenotypes should be prioritized in future studies aiming to investigate the biological effects of SHBG or develop targets for therapeutic intervention.

## Introduction

Sex hormone-binding globulin (SHBG) is a circulating glycoprotein functioning as a major transporter and putative regulator of androgens and estrogens (1). SHBG may also directly influence numerous traits and diseases independently of the hormones it regulates (2). Observational evidence has so far linked higher SHBG levels with lower risk of type 2 diabetes (3, 4), lower risk of prostate cancer (5) and higher risk of bone loss and fractures (6, 7). Genetic evidence supports the observed associations with type 2 diabetes (8, 9). Understanding the true scope of the impact that SHGB may have on the human phenome beyond type 2 diabetes is important since its molecular nature makes it, or the pathways it regulates, feasible targets for intervention (10–12).

SHBG levels are highly heritable (13, 14) with an estimated ~ 50% heritability in family studies. Genome-wide association studies (GWAS) have identified multiple polymorphisms at genome-wide significance that influence circulating SHBG in adult populations (15, 16), collectively explaining ∼15.6 and ∼8.4% of the heritable component of SHBG in men and women, respectively (15). These genetic underpinnings allow us to examine the causality of SHBG using Mendelian randomization (MR) methods that exploit the approximate randomization of germline genetic variants at conception (17, 18), to overcome the issues of confounding and reverse causation that are inherent to observational studies and limit causal inference.

**Figure 1:**
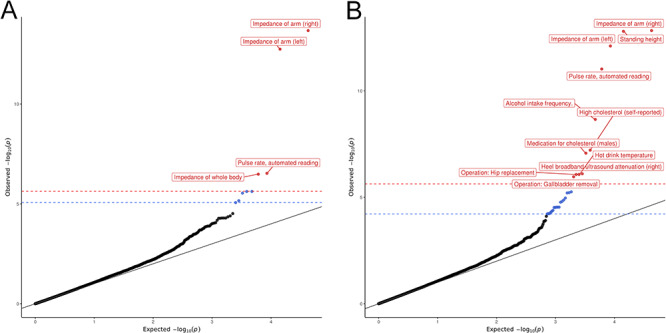
QQ plots of MR-pheWAS associations when using (**A**) an allele score comprised entirely of SNPs in the SHBG gene region (*cis*-SNPs) and (**B**) an allele score comprised of SNPs both within the SHBG gene region and on other chromosomes (*cis*-SNPs + *trans*-SNPs). Bonferroni corrected *P*-value threshold in red and FDR corrected *P*-value threshold in blue.

MR principles can also be applied in a hypothesis-free manner within a phenome-wide association study (pheWAS) approach called MR-pheWAS (19) to examine the effects of SHBG on thousands of measured traits and diseases, uncovering potentially causal associations that were not previously expected. The availability of large-scale data such as that from UK Biobank, a large UK-based prospective cohort(20, 21), means that phenome-wide scans can now provide sufficient statistical power to detect associations, since multiple testing burdens incurred are offset by large sample sizes. A recently published tool, the PHEnome Scan ANalysis Tool (PHESANT)(22), automates such scans in UK Biobank by passing available phenotypes through a rule-based sorting pipeline, categorizing all phenotypes in to either continuous, binary or ordinal outcomes. The automation offered by PHESANT enables systematic pheWAS across phenotypes that are highly heterogeneous.

We performed an MR-pheWAS using PHESANT to search for human traits and diseases that are potentially influenced by SHBG, using data from a large-scale cohort study (UK Biobank) which contains data on participants from clinical assessments, record linkage and health and lifestyle questionnaires. We then followed-up these findings using more formal two-sample MR methods to assess whether our results may be biased by horizontal pleiotropy, where the genetic instrument affects the outcome through pathways that are not via SHBG.

## Results

### MR-pheWAS

We tested the associations of both SHBG allele scores with 21 299 phenotypes in UK Biobank. For the 4-SNP *cis* allele score, there were four phenotype associations with a Bonferroni-adjusted *P*-value below 0.05 and a further five with FDR-adjusted *P*-value below 0.05 (Fig. 1A and Supplementary Table S3). For the 10-SNP *cis* + *trans* allele score, there were 11 phenotype associations with Bonferroni-adjusted *P*-value below 0.05 and a further 19 with FDR-adjusted *P*-value below 0.05 (Fig. 1B and Supplementary Table S4). All four phenotypes associated with the 4-SNP allele score below the Bonferroni-adjusted *P*-value threshold were also associated with the 10-SNP *cis* + *trans* allele score, below the FDR-adjusted *P*-value threshold. Specifically, the impedance of the right arm (*P* = 2.10 × 10^−14^), impedance of the left arm (*P* = 1.79 × 10^−13^), pulse rate (*P* = 2.95 × 10^−07^) and impedance of the whole body (*P* = 3.26 × 10^−07^) reached the Bonferroni-adjusted *P*-value threshold in the 4-SNP allele score. Overall in the MR-pheWAS, elevated SHBG was associated with body impedance, height, pulse rate, cholesterol levels, alcohol consumption, bone densitometry, hip replacement, gallbladder removal and hot drink temperature preference. Specifically, MR-pheWAS results for the 10 SNP *cis* + *trans* allele score indicated that higher genetically elevated SHBG was associated with a higher impedance of the right arm (*P* = 1.5 ×1 0^−13^), increased standing height (*P* = 1.6 × 10^−13^), higher impedance of left arm (*P* = 7.8 × 10^−13^), lower automated pulse rate reading (*P* = 9.4 × 10^−12^), lower alcohol intake frequency (*P* = 2.2 × 10^−9^), decreased odds of self-reported high-cholesterol (*P* = 6.1 × 10^−8^), decreased odds of medication for cholesterol in males (*P* = 8.5 × 10^−8^), decreased heel broadband ultrasound attenuation (right foot) (*P* = 2.2 × 10^−7^), decreased temperature preference for hot drinks (*P* = 8.4 × 10^−7^), increased odds of hip replacement operation (*P* = 2.2 × 10^−7^) and increased odds of gallbladder removal operation (*P* = 1.1 × 10^−6^). Impedance of whole body was associated with the 4-SNP allele score with a *P*-value (*P* = 1.4 × 10^−05^) below the Bonferroni threshold but just above the Bonferroni threshold for the 10-SNP *cis* + *trans* allele score although below the FDR threshold. Full results of the MR-pheWAS for both allele scores are shown in Supplementary Appendix A1.

In our sensitivity analysis for the 10-SNP *cis* + *trans* allele score adjusting for all 40 genetic PCs and genotype array, we observed only minimal differences with the results of the main MR-pheWAS for the 10-SNP *cis* + *trans* allele score. Effect estimates were identical with an *R*^2^ = 0.99 (Supplementary Fig. S3), and all phenotypes with a Bonferroni-adjusted *P*-value below 0.05 remained below 0.05 (results in Supplementary Table S5).

### Follow-up analysis

We followed-up all Bonferroni significant phenotypes identified in both our MR-pheWAS with two-sample MR to examine the heterogeneity of SNP effects and evidence of horizontal pleiotropy. Descriptive statistics are shown in Supplementary Tables S6–S8. We did not observe a low prevalence of cases for any of the binary or categorical phenotypes investigated, as shown in Supplementary Table S7.

### Two-sample MR

**Figure 2:**
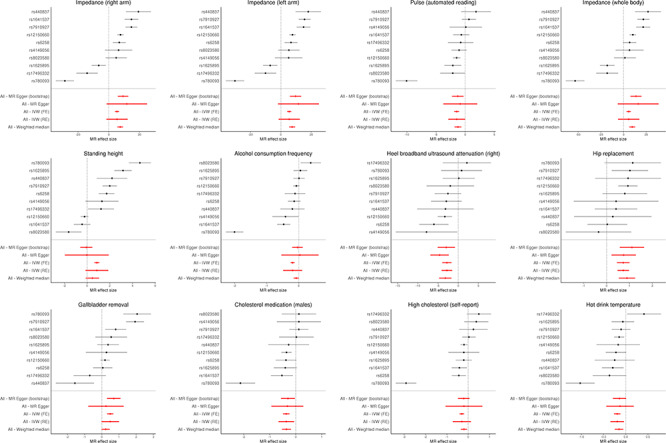
Forest plots of the two-sample MR results for phenotypes satisfying stringent correction for multiple testing (Bonferroni adjusted *P*-value <0.05) in the MR-pheWAS of SHBG, All-IVW (FE) = All-inverse variance weighted (fixed effects); All-IVW (RE) = All-inverse variance weighted (random effects). All MR effect sizes are per unit increase in natural log transformed SHBG (nmol/l). For phenotypes, impedance of right arm (units: ohms, field ID 23109), impedance of left arm (units: ohms, field ID 23110), pulse automated reading (units: beats-per-minute, field ID 102), impedance (whole body) (units: ohms, filed ID 23106), heel broadband ultrasound attenuation (right foot) (units dB/Mhz, field ID 4120), standing height (units: centimeters, field ID 50), alcohol consumption frequency (categorical intake, field ID 1558), hip replacement or revision (log odds, field ID 20004, value code #1318), gallbladder removal (cholecystectomy) (log odds, field ID 20004, value code #1455), cholesterol medication in males (log odds, field ID 6177, value code #1), self-report high cholesterol (log odds, field ID value 20002, code #1473) and hot drink temperature (categorical preference, field ID 1518).

Consistent direction of effects with overlapping confidence intervals (CIs) was observed between the five different MR estimation methods and the MR-pheWAS for all phenotypes below the Bonferroni *P*-value threshold except for standing height and alcohol consumption frequency (Fig. 2 and Supplementary Appendix A2). For impedance in right arm, left arm and whole body, hip replacement, pulse rate, heel broadband ultrasound attenuation and cholesterol medication in males, there was some evidence of an effect when using estimation methods partially or wholly robust to horizontal pleiotropy; the weighted median estimator and MR-Egger regression, with particularly strong evidence for an effect, observed for risk of hip replacement and heel broadband ultrasound attenuation. For gallbladder removal and self-reported high cholesterol, there was weaker evidence of an effect, with 95% CIs overlapping the null for estimated effects of the random-effects inverse variance weighted (IVW), weighted median and MR-Egger. The fixed-effects IVW indicated the associations of increased log SHBG with higher impedance of the right arm (5.36 [95% CI 4.0–6.7] ohms), lower pulse rate (−1.46 [95% CI −1.87, −1.0] beats-per-minute (bp)), higher impedance in the left arm (5.46 [95% CI 4.1–6.8] ohms), high impedance of the whole body (5.64 [95% CI 3.3–7.9] ohms), lower heel broadband ultrasound attenuation (right foot) (−2.8 [95% CI −4.0, −1.7] dB/Mhz), higher odds of hip replacement (OR = 2.1 [95% CI 1.6–2.7]), lower odds of taking cholesterol medication in males (OR = 0.69 [95% CI 0.6–0.8]), higher odds of gallbladder removal (OR = 1.63 [95% CI 1.4–2.0]), lower odds of self-report high cholesterol (OR = 0.74 [95% CI 0.66–0.83]) and decreased hot drink temperature preference (OR = 0.82 [95% CI 0.77–0.89]). Similarly, for the weighted median estimator, associations indicated higher impedance of the right arm (7.4 [95% CI 5.5–9.4] ohms), lower pulse rate (−1.2 [95% CI −1.8, −0.7] beats per minute (bp)), higher impedance in the left arm (7.4 [95% CI 5.5–9.3] ohms), high impedance of the whole body (9.5 [95% CI 6.1–12.9] ohms), lower heel broadband ultrasound attenuation (right foot) (−3.2 [95% CI −4.7, −1.7] dB/Mhz), higher odds of hip replacement (OR = 2.4 [95% CI 1.7–3.5]), lower odds of taking cholesterol medication in males (OR = 0.7 [95% CI 0.59–0.85]), higher odds of gallbladder removal (OR = 1.25 [95% CI 0.97–1.61]), lower odds of self-report high cholesterol (OR = 0.82 [95% CI 0.71–0.95]) and decreased hot drink temperature preference (OR = 0.86 [95% CI 0.78–0.95]). Full results for these phenotypes are presented in Supplementary Appendix A2.

Results of the sensitivity analysis, removing outliers from continuous phenotypes (more than four standard deviations from the mean), were consistent with the results of our main analysis, indicating that potential outliers have little effect on our results.

### Sensitivity analysis

We observed an extreme outlier among SNPs in our two-sample MR results. SNP rs780093 annotated to the *GCKR* gene gave the largest absolute effect estimate of SHBG on the majority of phenotypes followed-up, often an order of magnitude greater than that of the next SNP (seen in Fig. 2). The 95% CIs for the estimated effect of rs780093 on each phenotype did not overlap those of any of the other SNPs in most of the phenotypes followed-up at Bonferroni significance. Although the random-effects IVW takes between-SNP heterogeneity into account and the weighted median estimator is relatively robust to such outliers, fixed-effects IVW and MR Egger regression are not robust to extreme outliers. We therefore performed a sensitivity analysis, removing this variant to determine its impact on results. After removing this variant, our estimated effects on pulse rate, impedance (right arm, left arm and whole body), self-reported high cholesterol, cholesterol medication in males and hot drink temperature preference was a stronger evidence of an association with narrower CIs for some of the estimation methods used, while there was little impact for the other phenotypes (Supplementary Fig. S2 and Supplementary Appendix A2).

In addition to our sensitivity analysis excluding the *GCKR* genetic variant, we also conducted a further sensitivity analysis using an MR estimation method that evaluates horizontal pleiotropy in multi-instrument MR and corrects for horizontal pleiotropy via outlier removal, MR-PRESSO—Mendelian Randomization Pleiotropy RESidual Sum and Outlier (23). MR-PRESSO requires that at least 50% of genetic variants are valid IVs and subject to the InSIDE (instrument strength independent of direct effect) assumption.

There was no evidence that associations between genetically elevated SHBG and either heel broadband ultrasound attenuation (right foot) or risk of hip replacement were affected by outliers. Following correction for outliers, we observed the evidence of an association between genetically elevated SHBG and impedance in the right arm, impedance in the left arm, pulse rate, impedance of the whole body, standing height, cholesterol medication and hot drink temperature preference, but only weak evidence of an association with alcohol consumption frequency, gallbladder removal and self-report high cholesterol (Supplementary Table S9).

### Replication analyses

We searched the MR-Base repository (mrbase.org) for available outcome GWAS not conducted in UK Biobank to use for the replication of the associations identified in our MR-pheWAS. We identified only one such study, for pulse rate (heart rate, study #1056). The GWAS meta-analysis was conducted by den Hoed *et al.* (2013) in a sample of 92 355 individuals, comprised of both child and adult cohorts, of mostly white-European ancestry (24).

In our replication analysis, we found little evidence of association between SHBG and heart rate. Effect sizes were in a consistent direction to our discovery UK Biobank sample but with wide CIs that overlapped the null (Supplementary Fig. S5 and Table S10). There was a −0.6 [95% CI −1.30, 0.09] change in heart rate (bp) per unit increase in log SHBG for the IVW fixed effects and a −0.4 [95% CI −1.38, 0.55] change in heart rate (bp) for the weighted median estimator. CIs for all MR sensitivity analyses overlapped the null.

## Discussion

Our MR-pheWAS of genetically instrumented SHBG in UK Biobank suggested that SHBG influences multiple phenotypes. Our dual approach utilizing two allele scores, one more conservative which was comprised of SNPs solely in the *SHBG* gene region (which is less likely to be biased by pleiotropy) and one more liberal which was comprised of SNPs both in the *SHBG* gene region and outside it (which may be more prone to pleiotropic effects but explains a greater amount of variance in SHBG), indicated associations with higher body impedance, lower pulse rate, lower bone density, increased risk of hip replacement, gallbladder removal and high cholesterol, increased standing height and decreased alcohol intake frequency. Follow-up using two-sample MR indicated little evidence for an association of SHBG with alcohol consumption frequency and standing height, two phenotypes found using the more liberal allele score, but some evidence for all other phenotypes. In a replication analysis using publically available GWAS data and a two-sample MR approach, we were not able to replicate the novel association between SHBG and pulse rate. Overall, we identified two associations in the category of lifestyle and environment, five associations in the category of physical measures/anthropometry and four in the category of self-reported operations/medical conditions/medical history.

We did not perform a sex-stratified analysis as sex-specific summary statistics from the previously published GWAS of SHBG were not available for all genetic variants used as IVs. Specifically, 3 out of the 4 *cis* genetic variants, that constitute both of our allele scores and explain the greatest proportion of variance in SHBG, were identified in a whole-sample conditional analysis with no sex-specific effect sizes reported. Since a requirement of two-sample MR is that effects are derived from the same underlying populations, violating this assumption can result in bias (25). Future studies may aim to explore the sex-specific effects of SHBG in a two-sample MR framework by first deriving sex-specific results for all genetic variants associated with SHBG.

### Comparison to previous literature

#### Hip replacement, bone-mineral density and impedance

We observe associations between genetically elevated SHBG and two bone-related phenotypes, hip joint replacement and heel bone ultrasound attenuation (in the right foot) in the MR-pheWAS. Higher SHBG was associated with multiple measures of bone-densitometry in the MR-pheWAS (QQ plot in Supplementary Fig. S4) with *P*-values deviating substantially from those expected by chance in this category. Since bone densitometry in UK Biobank was performed on only a subsample of the full cohort, with the reduction of the resulting sample size for these measurements likely leading to reduced statistical power to detect effects, this may explain why the association with these phenotypes does not survive multiple testing adjustment despite having *P*-values lower than those expected by chance alone. Observational studies have linked higher SHBG to higher risk of hip replacement as well as of bone loss and fracture risk (6, 7), indicating that SHBG may contribute to bone mineral density (BMD) or bone strength. This supports previous genetic evidence of an effect of higher SHBG on higher BMD in a sample of men (26). Interestingly, joint replacement prevalence is also increased in individuals with high bone mass (27). Previous studies have suggested that estrogen levels (which are regulated by SHBG) control BMD in both men and women (28). Estrogen supplementation in women is often used to counter peri-menopausal symptoms such as the loss of BMD and changes in body composition, with some conflicting evidence indicating an association with increased SHBG (29–31). The likely complex relationship between SHBG and BMD, bone density/mass and risk of hip replacement warrants further investigation.

Associations with both impedance in the right and left arms, as well as whole body impedance, were observed in our analyses, suggesting an effect of higher SHBG on higher lean mass. Multiple other weaker associations with body composition were observed above the Bonferroni threshold but below the FDR threshold, including increased trunk fat mass (*P* = 6.2 × 10^−6^) and increased trunk fat percentage (*P* = 6 × 10^−6^), potentially indicating a systemic effect of SHBG on body impedance and composition. However, interestingly, there was no evidence of an association with BMI in the MR-pheWAS (*P* = 0.733). Several observational studies have investigated associations between SHBG and measures of body size and composition. A review of these studies focusing on postmenopausal women suggested an effect of lower SHBG on body composition, including lower BMI, waist circumference and waist-to-hip ratio, potentially through the accumulation of visceral fat (32). The same review also indicated an association with cardiovascular disease risk in women. Although we do not observe any strong associations with systolic or diastolic blood pressure in the MR-pheWAS, we did observe an association with lower pulse rate—a novel finding. However, we were unable to replicate the association between SHBG and pulse rate using publically available GWAS data in a follow-up MR replication step. Although we observed effect sizes in the same direction as our discovery analyses, CIs overlapped the null. The lack of replication may indicate that the novel association with pulse rate in the MR-pheWAS is a spurious finding. Notably, the replication dataset included the study participants of Asian ancestry (7% of total sample size), participants from a range of ages (mix of child and adult cohorts) and a range of pulse rate measurement methods (ECG, peripheral pulse rate or self-report), which may have biased results towards to the null if the association between SHBG and pulse rate differs by ancestry, age or measurement method.

#### Cholesterol, gallbladder removal and diabetes

A previous study suggested higher SHBG leads to a decrease in total cholesterol, VLDL cholesterol, IDL and LDL cholesterol at nominal significance levels (9), which is consistent with our own findings of lower risk of taking cholesterol medication (in males) and lower risk of self-reported high cholesterol. Associations with measured lipid fractions or total cholesterol from blood samples were not tested in our study as the data were not available in UK Biobank at the time of analysis.

We also observed an association between genetically elevated SHBG and higher odds of gallbladder removal in the MR-pheWAS. Gallbladder removal (cholecystectomy) is a common operative procedure for symptomatic gallstones which are predominantly composed of cholesterol and are common in individuals with high circulating cholesterol levels (33). Since lower SHBG was associated with increased self-reported high cholesterol and medication use, the results imply that if cholesterol levels are controlled using medication then individuals may also be at lower risk of gallbladder removal. Long-term use of statins for controlling cholesterol levels has been reported as associated with a decreased risk of gallstones followed by gallbladder removal (34, 35). It is possible that cholesterol-lowering medications (such as statins) may act as an effect moderator of the association of SHBG with gallbladder removal, with individuals with higher SHBG and therefore on cholesterol medication less likely to develop gallstones and have lower risk of gallbladder removal than the population average.

We observed little evidence of association between SHBG and self-reported doctors-diagnosis of diabetes (field ID 2443, *P* = 0.368) or self-reported type-2 diabetes (field ID 20002, value code #1223, *P* = 0.454) in the MR-pheWAS, despite prior observational and genetic evidence of a protective effect (8, 9, 36, 37). The effect estimate from the MR-pheWAS for the former phenotype was consistent with a protective effect of increased SHBG on diabetes, but with CIs that overlapped the null.

### Strengths and limitations

There are several major strengths of our study. First, using MR helps to reduce the potential that our results will be biased by confounding and reverse causation which limit the inference from observational studies. Second, the large sample size helps to offset the multiple testing burden of our hypothesis-free analysis. Third, the PHESANT’s automated rule-based approach is systematic and allows us to assess the effects of SHBG on a broad range of phenotypes, including those without existing hypothesized relationships with SHBG. Finally, since our exposure is a molecular phenotype, the genetic variants used in our allele score explain a relatively large proportion of the variance in the exposure (8–15% of the heritable component of SHBG), which is often not the case for MR studies where complex phenotypes are instrumented.

There are several limitations to our study. MR assumes a linear association between genetically elevated SHBG and the outcome, which may not be the case for all phenotypes we have analyzed. Similarly, SNP-outcome associations in UK Biobank were not adjusted for interactions between age and sex or for non-linear relations between age and risk of disease. IVs used in MR are considered a proxy for lifetime exposure and MR does not distinguish between age-specific effects of a potentially time-varying exposure such as SHBG. It is not possible to say whether the effects of elevated SHBG on the reported phenotypes are the results of exposure to elevated SHBG at particular times (e.g. if SHBG affects body composition wholly via timing of puberty, which leads to an effect on body composition which is thereafter fixed for life, then intervening on levels of SHBG in adulthood would not affect body composition). While this does not necessarily bias our results, it can have important implications when designing interventions. The MR-pheWAS approach uses a weighted allele score to assess the associations with phenotypes in UK Biobank, and if the genetic variants comprising the allele score are horizontally pleiotropic (i.e. they affect the phenotype through additional pathways that are not via circulating SHBG), then results may be biased. We have also not been able to assess the strength of our allele score or individual SNPs with SHBG in UK Biobank. The PHESANT’s automated approach may suboptimally pre-process some phenotypes, which may bias estimated effects. Measurement error in some UK Biobank phenotypes may be biasing results towards the null leading to fewer associations surviving our multiple testing threshold. Our hypothesis-free MR-pheWAS approach is also limited to the set of phenotypes recorded by UK Biobank at the time of conducting these analyses, which broadly encompasses outcomes (mostly from self-report) thought to be relevant to human health but may be missing several informative classes of data, particularly those that are more invasive or difficult to collect on a large cohort, such as measures from biological samples. The future application of MR-pheWAS to other cohorts that may have collected different data on participants may provide additional insight into the effects of SHBG on the human phenome. We did not impose any exclusions on possible phenotypes in the MR-pheWAS based on perceived biological relevance or from prior SHBG literature in order to avoid introducing researcher bias and maintain a systematic approach. This approach, however, likely inflates the multiple testing burden as many phenotypes (such as those relating to specific dietary habits) are likely uninformative to the effects of SHBG on health or may have a high level of redundancy**.** Our MR-pheWAS is therefore comprehensive to the available phenotypes in UK Biobank but not to the entire range of possible human health outcomes. Finally, the previous GWAS of SHBG from which SNP-SHBG associations were derived to calculate the allele score for the MR-pheWAS and two-sample MR was adjusted for BMI. This may introduce collider bias if BMI is an outcome of SHBG and some downstream outcome of SHBG.

We performed an MR-pheWAS in UK Biobank, to uncover potential causal effects of circulating SHBG. Our results suggest that higher circulating SHBG induces higher body impedance, lower bone-density, higher risk of hip replacement and gallbladder removal, and lower risk of high cholesterol. These phenotype associations should be prioritized for future studies aiming to investigate the effects of SHBG and to develop therapeutic interventions.

## Materials and Methods

### Study population

#### UK Biobank

UK Biobank is a UK-based prospective cohort of over 500 000 participants(20) aged 40–69 years at recruitment in 2006–2010. Recruitment details are described elsewhere (20, 38). Ethical approval was provided by the Research Ethics Committee (REC reference 11/NW/0382). Data on over 21 000 phenotypes are available as part of the study cohort, including anthropometric measurements, self-reported health conditions and data extracted from linked medical records (dataset 21 753, application 16 729). These phenotypes capture information on diet, physical activity, medical history, sociodemographics, psychosocial factors, overall lifestyle and environment as well as measures assessed in clinic such as bone densitometry, brain imaging and lung function, among others. The current data release used for this study does not contain measured biomarkers from biological samples such as blood (lipid fractions, circulating hormones, etc.). It also does not include information on participants from primary care (such as GP practices).

#### Genetic data

Genotypes in UK Biobank were assayed using two different arrays (chips), the Affymetrix UK BiLEVE Axiom or Affymetrix UK Biobank Axiom array. Genetic data pre-processing and sample exclusions have been previously described (39). Following exclusions, 334 977 individuals with genetic data remained.

#### SHBG score

A previous GWAS of SHBG (15) identified nine variants associated with circulating SHBG at genome-wide significance (discovery *N* = 21 791 mostly white European ancestry) in the main sample analysis. A further three independent ‘*cis*’ variants were identified in a conditional analysis of the *SHBG* gene locus. We used variants from the main GWAS analysis and those from the conditional analysis of the *SHBG* locus to derive SHBG allele scores, excluding only SNPs located on the X-chromosomes (rs1573036) and one SNP not available in the HRC-imputed UK Biobank dataset (rs2411984). We retained one SNP (rs780093) in the *GCKR* gene locus despite some prior evidence indicating pleiotropic effects (9). We instead investigated the possibility of horizontal pleiotropy at follow-up, rather than exclude *a priori*, which may lead to bias (40).

We used a dual approach to the MR-pheWAS by calculating two allele scores. The first allele score was comprised of four SNPs located in the *SHBG* gene region (*cis*-SNPs). The second allele score was comprised of SNPs in both the *SHBG* gene region and on other chromosomes (10 SNPs both *cis* + *trans*) as identified in the previous GWAS. Whereas genetic variants in the *SHBG* gene region may be considered more reliable proxies of SHBG as they are less likely to be subject to pleiotropy compared with variants outside the gene region, the more conservative 4-SNP *cis* allele score explains less of the variance in SHBG than the more liberal 10-SNP *cis* + *trans* allele score, which may lead to reduced power to detect effects. Both allele scores were calculated as the sum of the number of SHBG-increasing alleles (variant details with proportion of variance explained by each are provided in Supplementary Table S1), weighted by the effect sizes in the source GWAS (reported as natural log transformed nmol/l SHBG per allele), such that higher allele scores correspond to higher genetically determined SHBG. We used estimates from the combined discovery and replication meta-analysis of the GWAS conducted in both men and women, except for three variants in the *SHBG* gene locus, which were determined in a conditional analysis using only the discovery sample.

### Statistical analysis

#### MR-pheWAS

We performed an MR-pheWAS of UK Biobank phenotypes using PHESANT (v0.14) (22) in UK Biobank with an SHBG-increasing allele score as the exposure. Briefly, PHESANT uses rules to categorize each variable as one of four data types: continuous, ordered categorical, unordered categorical or binary. Inverse normal rank transformation is applied to the variables of the continuous data type to ensure a normal distribution. Outcomes classified as continuous that have fewer than 500 observations are excluded by default. Similarly, binary or categorical variables with fewer than 10 observations in any one category are excluded. A more detailed description of the process is available in Supplementary Methods. Certain variables relating mostly to cohort design (such assessment center), genetic data descriptor variables and age/sex of participants (used as covariates) are excluded from the pipeline. These *a priori* exclusions are listed in Supplementary Table S2. At the analysis stage, PHESANT estimates the univariate association of the SHBG allele score (the exposure) with each outcome variable. Outcome variables with continuous, binary, ordered categorical and unordered categorical data types are tested using linear, logistic, ordered logistic and multinomial logistic regression, respectively. All associations are adjusted for self-reported sex, age at time of assessment and the first 10 genetic principal components (PCs).

Since significance thresholds are largely arbitrary and cutoff thresholds should not be absolute (41, 42), we employed a heuristic approach by corrected for multiple testing using two different methods. First, we calculated a stringent Bonferroni-corrected *P*-value threshold, by dividing 0.05 by the number of tests performed, which assumes each test is independent. Second, we controlled for the expected proportion of false positive results among ‘hits’ using the false discovery rate (FDR). After ranking the results by *P*-value, we identify the largest rank position with a *P*-value less than *P*_threshold_ = 0.05 × rank/*n*, where *n* is the total number of tests and *P*_threshold_ is the *P*-value threshold resulting in a FDR of 5% (43).

As a sensitivity analysis, we reran the MR-pheWAS for the 10-SNP *cis* + *trans* allele score adjusting for the first 40 genetic PCs and the genotype array (chip) used, in addition to age and sex, to determine if population stratification or cryptic ancestry was affecting the results of the main MR-pheWAS.

### Follow-up

Phenotypes identified as associated with either SHBG allele score at a Bonferroni-adjusted *P*-value <0.05 in the MR pheWAS analysis were carried forward for formal two-sample MR analyses to assess heterogeneity between SHBG SNPs and whether estimated potentially causal effects may be biased due to horizontal pleiotropy. We applied the same genotype exclusions to the data as with the MR-pheWAS. We assessed each untransformed phenotype individually and decided on perceived normality of distributions (for continuous outcomes), presence of implausible outliers, adequate sample size for cases in binary variables (>500 cases) and which data type categorization (and hence analytical method) to apply in each case. We did not inverse-rank normalize the continuous phenotypes and preserve a more intuitive interpretation of effect estimates.

### Two-sample MR

We estimated the SNP-outcome effect in UK Biobank for all 10 SNPs comprising our allele scores and all continuous, binary and ordinal outcomes, followed-up using linear, logistic and ordinal logistic regression, respectively. Mirroring the main analysis of the MR-pheWAS, we adjusted associations for age, sex and first 10 genetic PCs. We used the same SNP-SHBG effects from the SHBG GWAS (i.e. the weights used for our SHBG allele score).

We used five complementary MR methods to estimate the effect of SHBG on each phenotype: fixed-effects IVW, random-effects IVW, the weighted median estimator, MR-Egger regression and MR-Egger regression with bootstrap standard errors. These methods have different strengths and assumptions. Respectively, they estimate effects that: are analogous to the weighted allele score in the MR-pheWAS (fixed effects IVW), take in to account heterogeneity between SNPs (random-effects IVW), are unbiased if up to 50% of the information from the IVs is invalid (weighted median) (44), are unbiased even if all IVs are invalid (MR-Egger regression) (45) under a weaker set of assumptions, and are unbiased even if all IVs are invalid but with increased power in some situations (MR Egger with bootstrap standard errors) (45) under a weaker set of assumptions. All two-sample MR analyses were run using the TwoSampleMR package (46) in R 3.5.0 RC.

As a sensitivity analysis for the continuous outcomes, we repeated our two-sample MR follow-up after removing potential outliers, defined as being more than four standard deviations from the mean, to determine if putative outliers in continuous phenotypes affect our results.

## Supplementary Material

Supplementary_Material_130519_ddz269

Supplementary_Appendix_A1_110519_ddz269

Supplementary_Appendix_A2_110519_ddz269

## Abbreviations

BMD: bone mineral density
FDR: false discovery rate
GWAS: genome wide association study
HRC: haplotype reference consortium
IDL: intermediate density lipoprotein
IVW: inverse variance weighted
LDL: low-density lipoprotein
MR: Mendelian randomization
OR: odds ratio
PCs: principal components
PHESANT: phenome scan analysis tool
pheWAS: phenome wide association study
QQ: quantile quantile
SHBG: sex hormone-binding globulin
SNP: single nucleotide polymorphisms
VLDL: very low-density lipoprotein
