# Supplementary material for: Impact of sex hormone-binding globulin on the human phenome
Source: Hum Mol Genet. 2020 Jan 10;29(11):1824–32. doi: 10.1093/hmg/ddz269 (PMC7372548; doi:10.1093/hmg/ddz269)
Supplement: Supplementary_Material_130519_ddz269 [file supplementary_material_130519_ddz269.docx]

# Supplementary Material

## Supplementary Methods

#### MR-pheWAS

The PHESANT pipeline and variable processing algorithm has been previously described in detail^(1)^. The process begins with the variable field type and uses rules to categorize each variable as one of four data types: continuous, ordered categorical, unordered categorical or binary. Variables with the continuous and integer field type are usually assigned to the continuous data type, but are assigned to ordered categorical if, for instance, there are only a few distinct values. Variables of the categorical (single) field type are assigned to either the binary, ordered categorical or unordered categorical, depending on whether the field has two distinct values, or has been specified as ordered or unordered in the PHESANT setup files. Variables of the categorical (multiple) field type are converted to a set of binary variables, one for each value in the categorical (multiple) fields. Outcomes classified as continuous that have fewer than 500 observations are excluded by default. Similarly, binary or categorical variables with fewer than 10 observations in any one category are excluded. Certain variables relating mostly to cohort design (such assessment centre), genetic data descriptor variables and age/sex of participants (used as covariates) are excluded from the pipeline as previously specified in the PHESANT description^(1)^ and indicated in Supplementary Table S2. Inverse normal rank transformation is applied to variables of the continuous data type, to ensure they are normally distributed.

## Supplementary Results

### MR-pheWAS

#### Sensitivity analysis

In the sensitivity analysis of the MR-pheWAS, which was adjusted additional for genotype array (chip) and top 40 PCs, two phenotypes that were analysed in the main analysis failed to run. The two phenotypes “light smokers, at least 100 smokes in lifetime” and “Country of Birth (non-UK)” failed to run at the analysis stage due to singularity in the model. Since there was no evidence of association for these two phenotypes in the main analysis, we did not follow these two phenotypes up further.

### Two-sample MR follow-up

#### Outlier sensitivity analysis

In a sensitivity analysis of the two-sample MR analysis we removed values from all continuous phenotypes if they were more than 4 standard deviations from the mean. We did not observe any substantial differences between these two-sample MR results and those of the main analysis indicating that any putative outliers are unlikely to be affecting interpretation of the results.


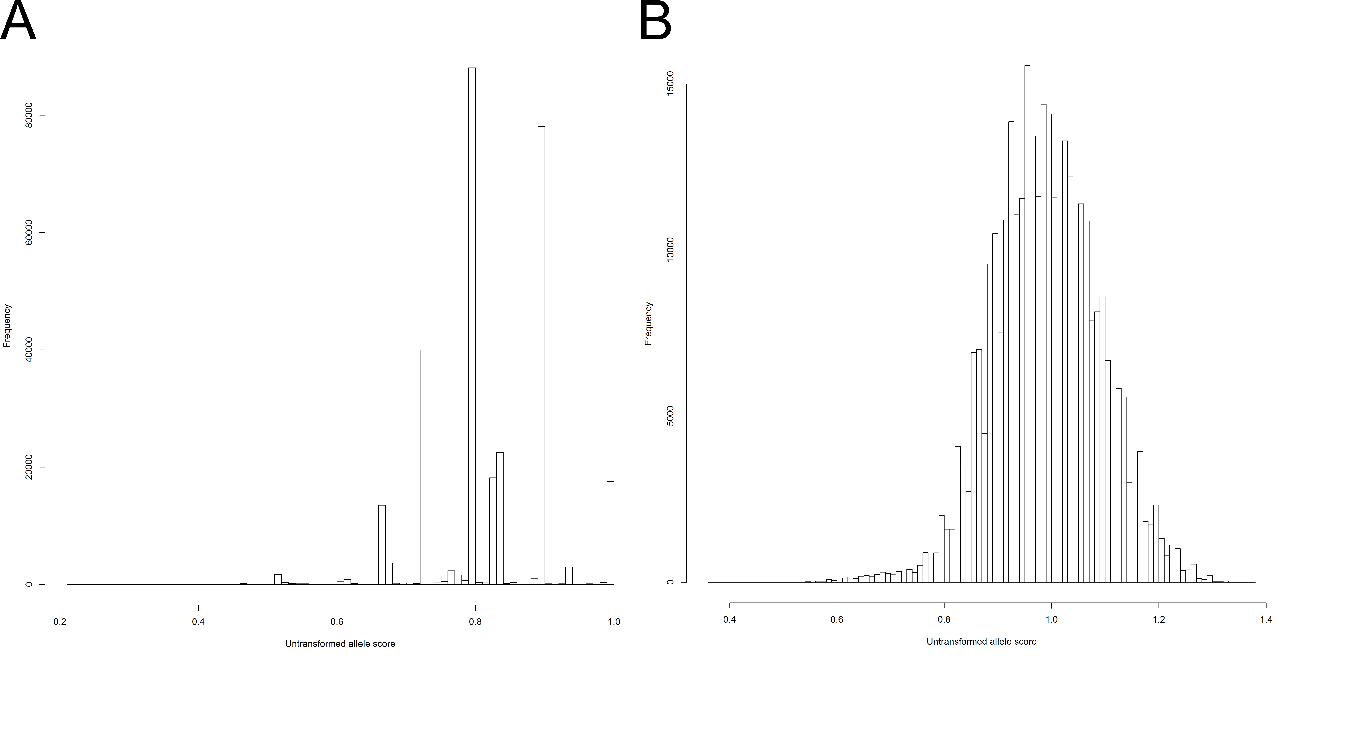


Figure S1 Histogram of SHBG allele scores, where A) cis allele score comprised of 4 SNPs in the SHBG gene region, and B) cis + trans allele score comprised of 10 SNPs both inside the SHBG gene region and on other chromosomes.


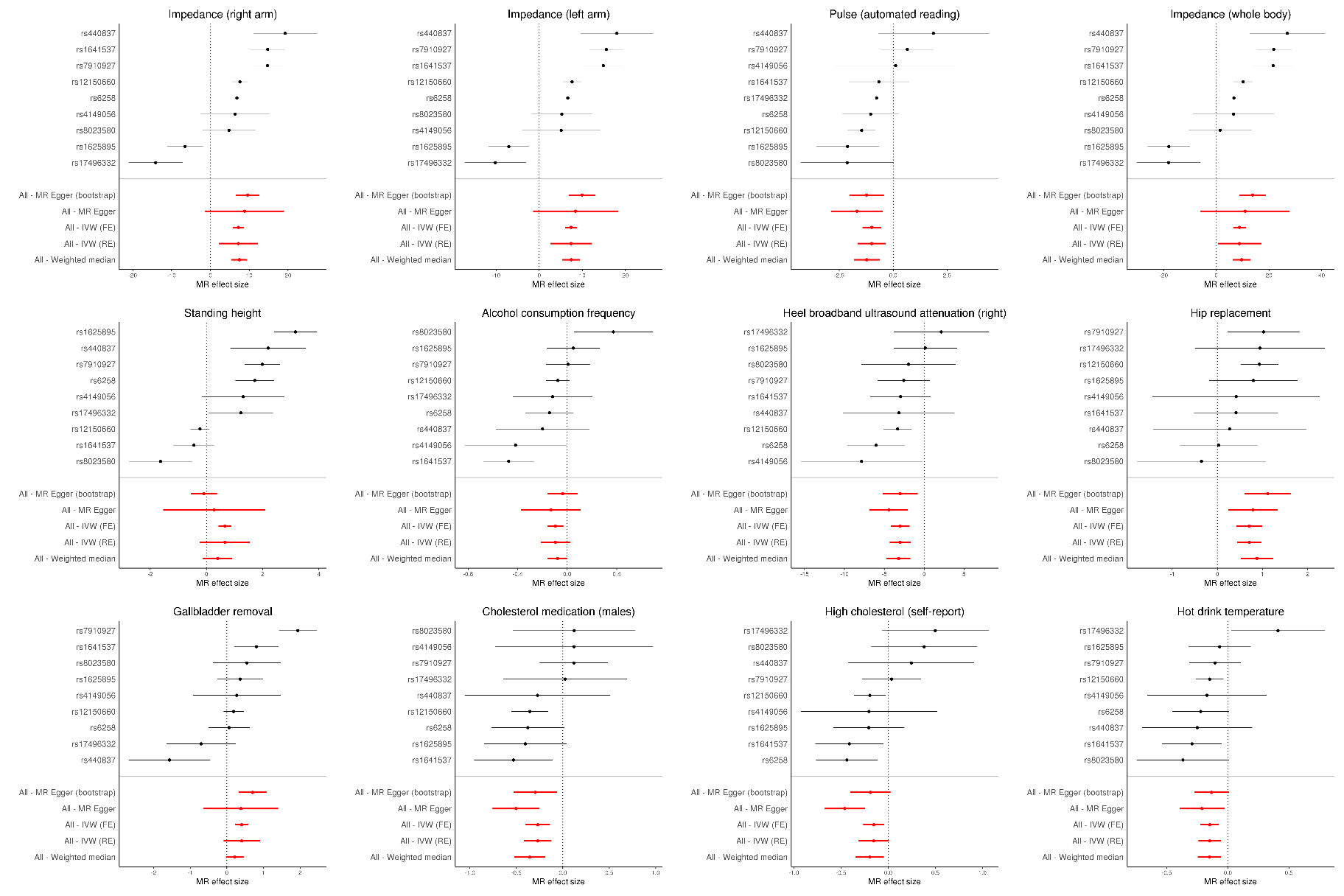


Figure S2 Forest plots of the two-sample MR sensitivity analysis excluding the rs780093 SNP for the phenotypes followed-up.


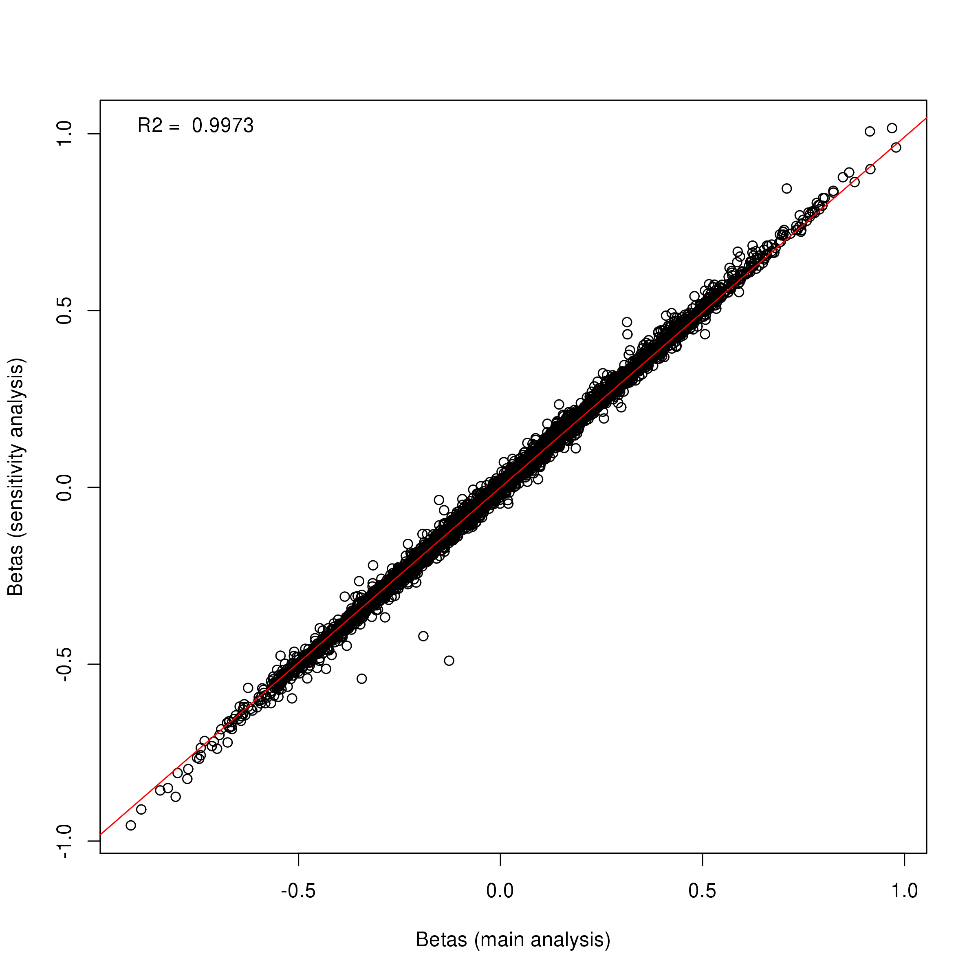


Figure S3 Comparison of betas for each phenotype between the main MR-pheWAS analysis for the cis + trans allele score and the MR-pheWAS sensitivity analysis for the same score (excluding multinomial logistic results).


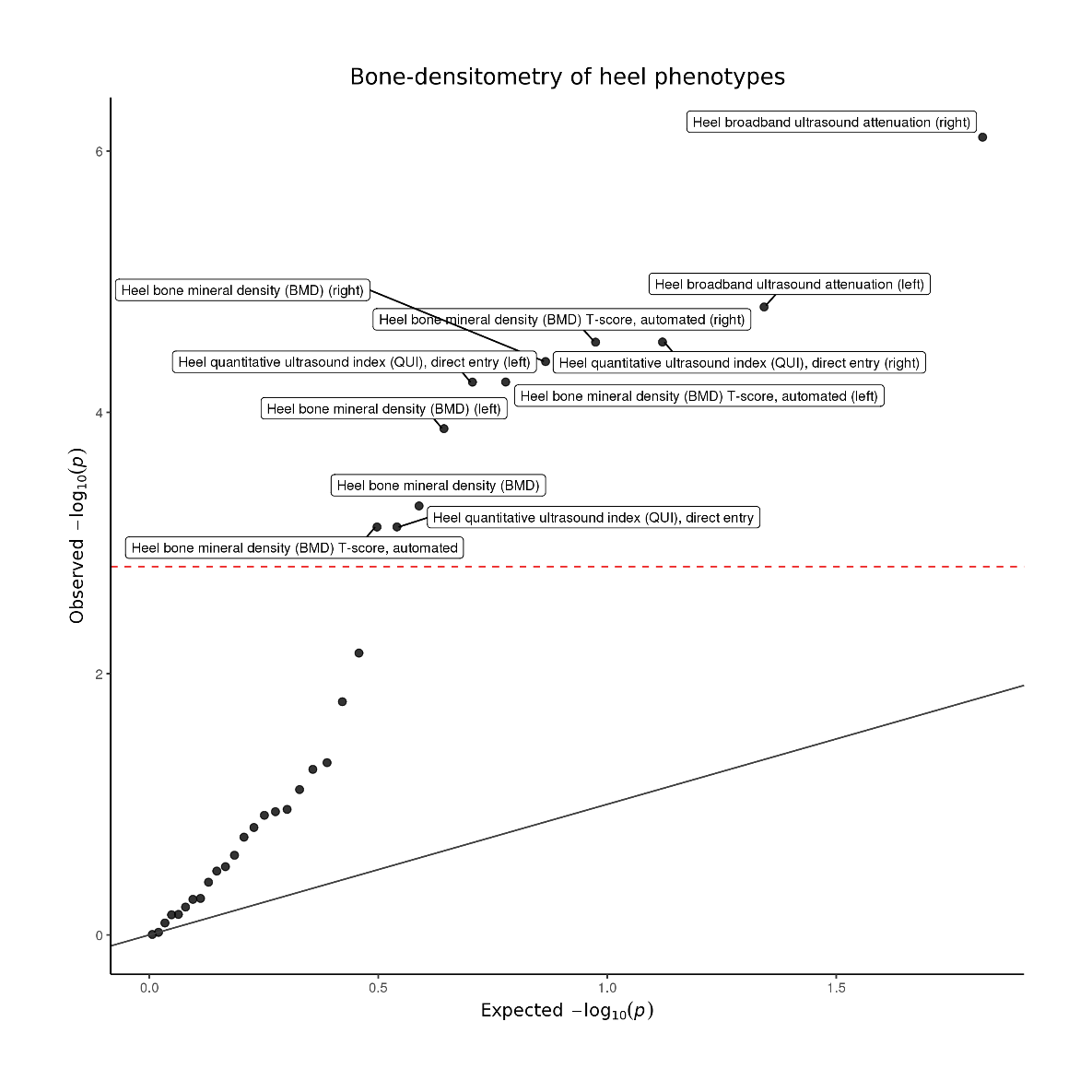


Figure S4 QQ plot of ‘Bone-densitometry of heel’ phenotypes (UK Biobank field category) for the MR-pheWAS of the cis allele score, with red dashed line indicating Bonferroni corrected threshold calculated for the number of phenotypes in the category only (0.05/33).


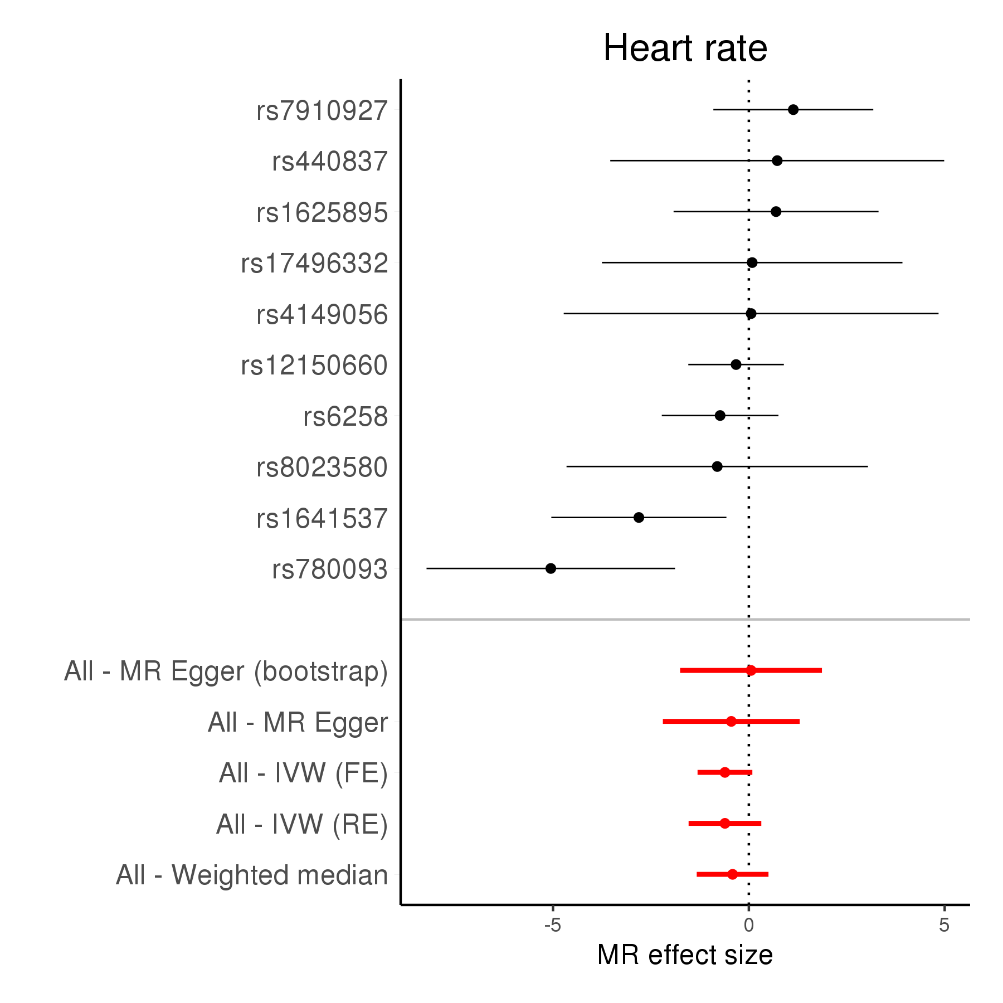


Figure S5 Forest plot of the two-sample MR replication analysis for heart rate, using publically available GWAS summary results in MR-Base.

Table S1 Reported summary statistics from the GWAS of SHBG by Coviello et al. used for the weighting of the allele scores and the two-sample MR follow-up.

| **SNP** | **Analysis‡** | **Region** | **Gene** | **Chr** | **Position** | **Effect** | **Other** | **EAF** | **Beta†** | **SE†** | **Pvalue†** | **PVE*** | **Allele score** |
| --- | --- | --- | --- | --- | --- | --- | --- | --- | --- | --- | --- | --- | --- |
| rs17496332 | Main | 1p13.3 | *PRMT6* | 1 | 1.07E+08 | a | g | 0.67 | -0.028 | 0.0041 | 1.40E-11 | 0.002136 | cis + trans |
| rs780093 | Main | 2p23.3 | *GCKR* | 2 | 27596107 | t | c | 0.4 | -0.032 | 0.0039 | 2.20E-16 | 0.00308 | cis + trans |
| rs440837 | Main | 8q21.13 | *ZBTB10* | 8 | 81624529 | a | g | 0.78 | -0.028 | 0.0047 | 3.40E-09 | 0.001626 | cis + trans |
| rs7910927 | Main | 10q21.3 | *JMJD1C* | 10 | 64808916 | t | g | 0.51 | -0.048 | 0.0039 | 6.10E-35 | 0.006903 | cis + trans |
| rs4149056 | Main | 12p12.1 | *SLCO1B1* | 12 | 21222816 | t | c | 0.82 | 0.029 | 0.0052 | 1.90E-08 | 0.001425 | cis + trans |
| rs8023580 | Main | 15q26.2 | *NR2F2* | 15 | 94509295 | t | c | 0.72 | -0.03 | 0.0044 | 8.30E-12 | 0.002129 | cis + trans |
| rs12150660 | Main | 17p13.1 | *SHBG* | 17 | 7462640 | t | g | 0.24 | 0.103 | 0.0047 | 1.80E-106 | 0.021564 | cis only, cis + trans |
| rs1641537 | Indep | 17p13.1 | *SHBG* | 17 | 7486446 | t | c | 0.14 | -0.064 | 0.006 | 1.20E-24 | 0.005194 | cis only, cis + trans |
| rs1625895 | Indep | 17p13.1 | *SHBG* | 17 | 7518840 | t | c | 0.12 | -0.06 | 0.006 | 1.75E-21 | 0.004568 | cis only, cis + trans |
| rs6258 | Indep | 17p13.1 | *SHBG* | 17 | 7475402 | t | c | 0.02 | -0.272 | 0.017 | 1.03E-60 | 0.011612 | cis only, cis + trans |

**PVE – Proportion of variance explained for each SNP, calculated based on reported summary statistics and the formula by Shim et al.^(2)^*

*†Effect estimate, standard error and p-value from the combined discovery plus follow-up analysis by Coviello et al.^(3)^ for the whole sample, except for the independent SNP analysis (Indep) where only the discovery sample was used.*

*‡Main GWAS analysis by Coviello et al. or independent SNPs analysis of the SHBG gene region.*

Table S2 Fields in UK Biobank that were excluded a priori from the MR-pheWAS.

| **Measure** | **Measure ID** | **Category** | **FieldID** | **UK Biobank field identifier** | **Trait** | **Units** |
| --- | --- | --- | --- | --- | --- | --- |
| UK Biobank Assessment Centre | 100024 | Recruitment | NA | 53 | Date of attending assessment centre | Date |
| Population characteristics | 100094 | Baseline characteristics | Baseline characteristics | 31 | Sex | Categorical single |
| Population characteristics | 100094 | Baseline characteristics | Baseline characteristics | 34 | Year of birth | Integer |
| Population characteristics | 100094 | Baseline characteristics | Baseline characteristics | 52 | Month of birth | Categorical single |
| UK Biobank Assessment Centre | 100021 | Recruitment | Reception | 54 | UK Biobank assessment centre | Categorical single |
| UK Biobank Assessment Centre | 100071 | Verbal interview | Medical conditions | 87 | Non-cancer illness year/age first occurred | Integer |
| UK Biobank Assessment Centre | 100071 | Verbal interview | Operations | 92 | Operation year/age first occurred | Integer |
| UK Biobank Assessment Centre | 100006 | Physical measures | Spirometry | 3059 | Result ranking | Categorical single |
| UK Biobank Assessment Centre | 100006 | Physical measures | Spirometry | 3065 | Ordering of blows | Categorical single |
| UK Biobank Assessment Centre | 100006 | Physical measures | Bone-densitometry of heel | 3081 | Foot measured for bone density | Categorical single |
| UK Biobank Assessment Centre | 100006 | Physical measures | Hearing test | 4232 | Triplet correct (left) | Categorical single |
| UK Biobank Assessment Centre | 100006 | Physical measures | Hearing test | 4243 | Triplet correct (right) | Categorical single |
| UK Biobank Assessment Centre | 100026 | Cognitive function | Numeric memory test | 4259 | Digits entered correctly | Categorical single |
| UK Biobank Assessment Centre | 100006 | Physical measures | Hearing test | 4268 | Completion status (left) | Categorical single |
| UK Biobank Assessment Centre | 100006 | Physical measures | Hearing test | 4275 | Completion status (right) | Categorical single |
| UK Biobank Assessment Centre | 100026 | Cognitive function | Numeric memory test | 4281 | Completion status of numeric memory test | Categorical single |
| UK Biobank Assessment Centre | 100026 | Cognitive function | Prospective memory test | 4287 | Test completion status | Categorical single |
| UK Biobank Assessment Centre | 100006 | Physical measures | Eye measures | 5090 | Refractometry result unreliable (left) | Categorical single |
| UK Biobank Assessment Centre | 100006 | Physical measures | Eye measures | 5091 | Refractometry result unreliable (right) | Categorical single |
| UK Biobank Assessment Centre | 100006 | Physical measures | Eye measures | 5136 | 3mm keratometry result unreliable (left) | Categorical single |
| UK Biobank Assessment Centre | 100006 | Physical measures | Eye measures | 5138 | 6mm keratometry result unreliable (left) | Categorical single |
| UK Biobank Assessment Centre | 100006 | Physical measures | Eye measures | 5139 | 6mm keratometry result unreliable (right) | Categorical single |
| UK Biobank Assessment Centre | 100006 | Physical measures | Eye measures | 5140 | 3mm keratometry result unreliable (right) | Categorical single |
| UK Biobank Assessment Centre | 100006 | Physical measures | Eye measures | 5141 | 3mm asymmetry index unreliable (left) | Categorical single |
| UK Biobank Assessment Centre | 100006 | Physical measures | Eye measures | 5142 | 6mm asymmetry index unreliable (left) | Categorical single |
| UK Biobank Assessment Centre | 100006 | Physical measures | Eye measures | 5143 | 6mm asymmetry index unreliable (right) | Categorical single |
| UK Biobank Assessment Centre | 100006 | Physical measures | Eye measures | 5144 | 3mm asymmetry index unreliable (right) | Categorical single |
| UK Biobank Assessment Centre | 100006 | Physical measures | Eye measures | 5145 | 3mm regularity index unreliable (right) | Categorical single |
| UK Biobank Assessment Centre | 100006 | Physical measures | Eye measures | 5146 | 6mm regularity index unreliable (right) | Categorical single |
| UK Biobank Assessment Centre | 100006 | Physical measures | Eye measures | 5147 | 6mm regularity index unreliable (left) | Categorical single |
| UK Biobank Assessment Centre | 100006 | Physical measures | Eye measures | 5148 | 3mm regularity index unreliable (left) | Categorical single |
| UK Biobank Assessment Centre | 100006 | Physical measures | Eye measures | 5149 | 3mm regularity index for irregular astigmatism level (left) | Categorical single |
| UK Biobank Assessment Centre | 100006 | Physical measures | Eye measures | 5152 | 3mm asymmetry index for irregular astigmatism level (right) | Categorical single |
| UK Biobank Assessment Centre | 100006 | Physical measures | Eye measures | 5155 | 3mm asymmetry index for irregular astigmatism level (left) | Categorical single |
| UK Biobank Assessment Centre | 100006 | Physical measures | Eye measures | 5164 | 3mm regularity index for irregular astigmatism level (right) | Categorical single |
| UK Biobank Assessment Centre | 100006 | Physical measures | ECG during exercise | 6024 | Program category | Categorical single |
| UK Biobank Assessment Centre | 100006 | Physical measures | Eye measures | 6074 | Glasses worn/required (right) | Categorical single |
| UK Biobank Assessment Centre | 100006 | Physical measures | Eye measures | 6075 | Glasses worn/required (left) | Categorical single |
| UK Biobank Assessment Centre | 100006 | Physical measures | Spirometry | 10691 | Result ranking (pilot) | Categorical single |
| UK Biobank Assessment Centre | 100071 | Verbal interview | Medical conditions | 20012 | Method of recording time when cancer first diagnosed | Categorical single |
| UK Biobank Assessment Centre | 100071 | Verbal interview | Medical conditions | 20013 | Method of recording time when non-cancer illness first diagnosed | Categorical single |
| UK Biobank Assessment Centre | 100071 | Verbal interview | Operations | 20014 | Method of recording time when operation occurred | Categorical single |
| UK Biobank Assessment Centre | 100021 | Recruitment | Reception | 21003 | Age when attended assessment centre | Integer |
| Population characteristics | 100094 | Baseline characteristics | Baseline characteristics | 21022 | Age at recruitment | Integer |
| Genomics | 100318 | Genotyping process | Genotyping process | 22000 | Genotype measurement batch | Categorical single |
| Genomics | 100314 | Genomics | Genomics | 22001 | Genetic sex | Categorical single |
| Genomics | 100314 | Genomics | Genomics | 22003 | Heterozygosity | Continuous |
| Genomics | 100314 | Genomics | Genomics | 22004 | Heterozygosity | Ongoing |
| Genomics | 100314 | Genomics | Genomics | 22005 | Missingness | Continuous |
| Genomics | 100314 | Genomics | Genomics | 22006 | Genetic ethnic grouping | Categorical single |
| Genomics | 100314 | Genomics | Genomics | 22009 | Genetic principal components | Continuous |
| Genomics | 100314 | Genomics | Genomics | 22010 | Recommended genomic analysis exclusions | Categorical single |
| Genomics | 100314 | Genomics | Genomics | 22011 | Genetic relatedness pairing | Integer |
| Genomics | 100314 | Genomics | Genomics | 22012 | Genetic relatedness factor | Continuous |
| Genomics | 100314 | Genomics | Genomics | 22013 | Genetic relatedness IBS0 | Continuous |
| Genomics | 100317 | Genotyping intensities | Genotyping intensities | 22014 | Average X chromosome intensities for determining sex | Continuous |
| Genomics | 100317 | Genotyping intensities | Genotyping intensities | 22015 | Average Y chromosome intensities for determining sex | Continuous |
| Genomics | 100318 | Genotyping process | Genotyping process | 22050 | UKBiLEVE Affymetrix quality control for samples | Categorical single |
| Genomics | 100318 | Genotyping process | Genotyping process | 22051 | UKBiLEVE genotype quality control for samples | Categorical single |
| Genomics | 100314 | Genomics | Genomics | 22052 | UKBiLEVE unrelatedness indicator | Categorical single |
| Genomics | 199001 | Interim genotype release | Interim genotype release | 22018 | Genetic relatedness exclusions | Categorical single |
| Genomics | 100313 | Genotyping process and sample QC | Genotyping process and sample QC | 22019 | Sex chromomosome aneuploidy | Categorical single |
| Genomics | 100313 | Genotyping process and sample QC | Genotyping process and sample QC | 22020 | Used in genetic principal components | Categorical single |
| Genomics | 100313 | Genotyping process and sample QC | Genotyping process and sample QC | 22021 | Genetic kinship to other participants | Categorical single |
| Genomics | 100313 | Genotyping process and sample QC | Genotyping process and sample QC | 22022 | Sex inference X probe-intensity | Continuous |
| Genomics | 100313 | Genotyping process and sample QC | Genotyping process and sample QC | 22023 | Sex inference Y probe-intensity | Continuous |
| Genomics | 100313 | Genotyping process and sample QC | Genotyping process and sample QC | 22024 | DNA concentration | Continuous |
| Genomics | 100313 | Genotyping process and sample QC | Genotyping process and sample QC | 22025 | Affymetrix quality control metric Cluster.CR | Continuous |
| Genomics | 100313 | Genotyping process and sample QC | Genotyping process and sample QC | 22026 | Affymetrix quality control metric dQC | Continuous |
| Genomics | 100313 | Genotyping process and sample QC | Genotyping process and sample QC | 22027 | Outliers for heterozygosity or missing rate | Categorical single |
| Genomics | 100313 | Genotyping process and sample QC | Genotyping process and sample QC | 22028 | Use in phasing Chromosomes 1-22 | Categorical single |
| Genomics | 100313 | Genotyping process and sample QC | Genotyping process and sample QC | 22029 | Use in phasing Chromosome X | Categorical single |
| Genomics | 100313 | Genotyping process and sample QC | Genotyping process and sample QC | 22030 | Use in phasing Chromosome XY | Categorical single |
| Online follow-up | 123 | Work environment | Employment history | 22200 | Year of birth | Integer |
| UK Biobank Assessment Centre | 100021 | Recruitment | Consent | 393 | Program (tactus) version ID (compiler timestamp) | Categorical single |
| Additional exposures | 1008 | Physical activity measurement | Raw accelerometer statistics | 90179 | Device ID | Integer |

Table S3 Results of MR-pheWAS for the 4 SNP cis allele score for phenotypes with an FDR-adjusted p-value <0.05.

| **UK Biobank field identifier** | **N** | **beta** | **Lower CI** | **Upper CI** | **pvalue** | **Analytical method** | **Phenotype** | **Measure** | **Category** | **FDR** | **Bonf** |
| --- | --- | --- | --- | --- | --- | --- | --- | --- | --- | --- | --- |
| 23109 | 329059 | 0.009313 | 0.006925 | 0.011701 | 2.10E-14 | Linear | Impedance of arm (right) | Physical measures | Anthropometry | 4.48E-10 | 4.48E-10 |
| 23110 | 329070 | 0.009 | 0.006605 | 0.011396 | 1.79E-13 | Linear | Impedance of arm (left) | Physical measures | Anthropometry | 1.91E-09 | 3.82E-09 |
| 102 | 315733 | -0.00907 | -0.01254 | -0.0056 | 2.95E-07 | Linear | Pulse rate, automated reading | Physical measures | Blood pressure | 0.001736 | 0.006286 |
| 23106 | 329066 | 0.006744 | 0.004156 | 0.009331 | 3.26E-07 | Linear | Impedance of whole body | Physical measures | Anthropometry | 0.001736 | 0.006943 |
| 4120 | 105633 | -0.01371 | -0.01941 | -0.00802 | 2.38E-06 | Linear | Heel broadband ultrasound attenuation (right) | Physical measures | Bone-densitometry of heel | 0.00851 | 0.050773 |
| 41204#D376 | 334945/23(334968) | -0.75601 | -1.05149 | -0.42217 | 2.40E-06 | Logistic-binary | Diagnoses - secondary ICD10 | Hospital in-patient | Diagnoses | 0.00851 | 0.051058 |
| 41210#C692 | 334921/47(334968) | -0.58263 | -0.81648 | -0.32922 | 2.89E-06 | Logistic-binary | Operative procedures - secondary OPCS | Hospital in-patient | Operations | 0.008793 | 0.061552 |
| 6177#1 | 117994/35343(153337) | -0.02858 | -0.04104 | -0.01612 | 6.93E-06 | Logistic-binary | Medication for cholesterol, blood pressure or diabetes | Touchscreen | Health and medical history | 0.018459 | 0.147673 |
| 23127 | 328890 | 0.006975 | 0.003903 | 0.010047 | 8.61E-06 | Linear | Trunk fat percentage | Physical measures | Anthropometry | 0.020378 | 0.1834 |

Table S4 Results of MR-pheWAS of the 10 SNP cis + trans allele score for phenotypes with an FDR-adjusted p-value <0.05.

| **UK Biobank variable identifier** | **N** | **beta** | **Lower CI** | **Upper CI** | **pvalue** | **Analytical method** | **Phenotype** | **Measure** | **Category** | **FDR** | **Bonf** |
| --- | --- | --- | --- | --- | --- | --- | --- | --- | --- | --- | --- |
| 23109 | 329059 | 0.009005 | 0.006616 | 0.011393 | 1.47E-13 | Linear | Impedance of arm (right) | Physical measures | Anthropometry | 1.70E-09 | 3.13E-09 |
| 50 | 334232 | 0.008939 | 0.006565 | 0.011314 | 1.59E-13 | Linear | Standing height | Physical measures | Anthropometry | 1.70E-09 | 3.39E-09 |
| 23110 | 329070 | 0.008759 | 0.006363 | 0.011155 | 7.77E-13 | Linear | Impedance of arm (left) | Physical measures | Anthropometry | 5.52E-09 | 1.65E-08 |
| 102 | 315733 | -0.01206 | -0.01553 | -0.00859 | 9.38E-12 | Linear | Pulse rate, automated reading | Physical measures | Blood pressure | 5.00E-08 | 2.00E-07 |
| 1558 | 334735 | -0.01838 | -0.0244 | -0.01236 | 2.19E-09 | Ordered-logistic | Alcohol intake frequency. | Touchscreen | Lifestyle and environment | 9.31E-06 | 4.66E-05 |
| 20002#1473 | 294067/40860(334927) | -0.02935 | -0.03997 | -0.01873 | 6.11E-08 | Logistic-binary | Non-cancer illness code, self-reported | Verbal interview | Medical conditions | 0.000217 | 0.001301 |
| 6177#1 | 117994/35343(153337) | -0.03408 | -0.04654 | -0.02161 | 8.45E-08 | Logistic-binary | Medication for cholesterol, blood pressure or diabetes | Touchscreen | Health and medical history | 0.000257 | 0.001799 |
| 4120 | 105633 | -0.01435 | -0.02004 | -0.00866 | 7.83E-07 | Linear | Heel broadband ultrasound attenuation (right) | Physical measures | Bone-densitometry of heel | 0.001803 | 0.016685 |
| 1518 | 331476 | -0.01799 | -0.02515 | -0.01083 | 8.42E-07 | Ordered-logistic | Hot drink temperature | Touchscreen | Lifestyle and environment | 0.001803 | 0.017928 |
| 20004#1318 | 329526/5401(334927) | 0.068075 | 0.040989 | 0.095179 | 8.47E-07 | Logistic-binary | Operation code | Verbal interview | Operations | 0.001803 | 0.018033 |
| 20004#1455 | 321660/13267(334927) | 0.043669 | 0.02612 | 0.061227 | 1.08E-06 | Logistic-binary | Operation code | Verbal interview | Operations | 0.002095 | 0.023048 |
| 20153 | 105066 | 0.00595 | 0.003383 | 0.008517 | 5.53E-06 | Linear | Forced expiratory volume in 1-second (FEV1), predicted | Physical measures | Spirometry | 0.009492 | 0.117888 |
| 23127 | 328890 | 0.007096 | 0.004023 | 0.010169 | 6.01E-06 | Linear | Trunk fat percentage | Physical measures | Anthropometry | 0.009492 | 0.128023 |
| 23128 | 328871 | 0.007832 | 0.004435 | 0.01123 | 6.24E-06 | Linear | Trunk fat mass | Physical measures | Anthropometry | 0.009492 | 0.132882 |
| 41200#W381 | 331620/3348(334968) | 0.076808 | 0.042608 | 0.111037 | 1.08E-05 | Logistic-binary | Operative procedures - main OPCS | Hospital in-patient | Operations | 0.015376 | 0.230642 |
| 23106 | 329066 | 0.005747 | 0.003159 | 0.008335 | 1.35E-05 | Linear | Impedance of whole body | Physical measures | Anthropometry | 0.017916 | 0.28665 |
| 4101 | 105662 | -0.01262 | -0.01834 | -0.00689 | 1.56E-05 | Linear | Heel broadband ultrasound attenuation (left) | Physical measures | Bone-densitometry of heel | 0.019566 | 0.332625 |
| 41200#L671 | 334548/420(334968) | -0.20691 | -0.30088 | -0.11232 | 1.70E-05 | Logistic-binary | Operative procedures - main OPCS | Hospital in-patient | Operations | 0.020163 | 0.362938 |
| 4123 | 105639 | -0.01242 | -0.01825 | -0.0066 | 2.90E-05 | Linear | Heel quantitative ultrasound index (QUI), direct entry (right) | Physical measures | Bone-densitometry of heel | 0.027809 | 0.617003 |
| 41204#D376 | 334945/23(334968) | -0.75122 | -1.08735 | -0.38468 | 2.90E-05 | Logistic-binary | Diagnoses - secondary ICD10 | Hospital in-patient | Diagnoses | 0.027809 | 0.617881 |
| 4125 | 105639 | -0.01242 | -0.01824 | -0.0066 | 2.90E-05 | Linear | Heel bone mineral density (BMD) T-score, automated (right) | Physical measures | Bone-densitometry of heel | 0.027809 | 0.618333 |
| 41221#7 | 6272/1025(7297) | 0.142528 | 0.075719 | 0.209598 | 3.00E-05 | Logistic-binary | Delivery methods | Hospital in-patient | Maternity | 0.027809 | 0.638544 |
| 2139 | 295123 | 0.007458 | 0.003955 | 0.01096 | 3.00E-05 | Linear | Age first had sexual intercourse | Touchscreen | Lifestyle and environment | 0.027809 | 0.6396 |
| 4124 | 105582 | -0.01219 | -0.01801 | -0.00636 | 4.09E-05 | Linear | Heel bone mineral density (BMD) (right) | Physical measures | Bone-densitometry of heel | 0.036262 | 0.87029 |
| 20004#1458 | 295440/39487(334927) | -0.02191 | -0.03243 | -0.01138 | 4.51E-05 | Logistic-binary | Operation code | Verbal interview | Operations | 0.038435 | 0.960878 |
| 2375 | 149748 | 0.026392 | 0.013644 | 0.039141 | 4.96E-05 | Ordered-logistic | Relative age of first facial hair | Touchscreen | Sex-specific factors | 0.040626 | 1 |
| 1528 | 308989 | -0.01358 | -0.02019 | -0.00697 | 5.65E-05 | Ordered-logistic | Water intake | Touchscreen | Lifestyle and environment | 0.043052 | 1 |
| 4106 | 105665 | -0.01198 | -0.01782 | -0.00614 | 5.86E-05 | Linear | Heel bone mineral density (BMD) T-score, automated (left) | Physical measures | Bone-densitometry of heel | 0.043052 | 1 |
| 4104 | 105665 | -0.01198 | -0.01782 | -0.00614 | 5.87E-05 | Linear | Heel quantitative ultrasound index (QUI), direct entry (left) | Physical measures | Bone-densitometry of heel | 0.043052 | 1 |
| 3581 | 103598 | -0.01226 | -0.01825 | -0.00627 | 6.06E-05 | Linear | Age at menopause (last menstrual period) | Touchscreen | Sex-specific factors | 0.043052 | 1 |

Table S5 Results of MR-pheWAS sensitivity analysis for the 10 SNP cis + trans allele score.

| **UK Biobank variable identifier** | **N** | **beta** | **Lower CI** | **Upper CI** | **pvalue** | **Analytical method** | **Phenotype** | **Measure** | **Category** | **FDR** | **Bonf** |
| --- | --- | --- | --- | --- | --- | --- | --- | --- | --- | --- | --- |
| 50 | 334232 | 0.009114 | 0.006745 | 0.011484 | 4.74E-14 | Linear | Standing height | Physical measures | Anthropometry | 1.01E-09 | 1.01E-09 |
| 23109 | 329059 | 0.009014 | 0.006626 | 0.011402 | 1.37E-13 | Linear | Impedance of arm (right) | Physical measures | Anthropometry | 1.46E-09 | 2.92E-09 |
| 23110 | 329070 | 0.008766 | 0.006371 | 0.011162 | 7.36E-13 | Linear | Impedance of arm (left) | Physical measures | Anthropometry | 5.22E-09 | 1.57E-08 |
| 102 | 315733 | -0.01201 | -0.01548 | -0.00854 | 1.12E-11 | Linear | Pulse rate, automated reading | Physical measures | Blood pressure | 5.99E-08 | 2.39E-07 |
| 1558 | 334735 | -0.01826 | -0.02428 | -0.01224 | 2.81E-09 | Ordered-logistic | Alcohol intake frequency. | Touchscreen | Lifestyle and environment | 1.2E-05 | 5.98E-05 |
| 20002#1473 | 294067/40860(334927) | -0.02981 | -0.04044 | -0.01918 | 3.85E-08 | Logistic-binary | High cholesterol (self-reported) | Verbal interview | Medical conditions | 0.000137 | 0.00082 |
| 6177#1 | 117994/35343(153337) | -0.03412 | -0.0466 | -0.02164 | 8.33E-08 | Logistic-binary | Medication for cholesterol (males) | Touchscreen | Health and medical history | 0.000253 | 0.001774 |
| 4120 | 105633 | -0.0144 | -0.02009 | -0.00871 | 7.15E-07 | Linear | Heel broadband ultrasound attenuation (right) | Physical measures | Bone-densitometry of heel | 0.001904 | 0.01523 |
| 20004#1318 | 329526/5401(334927) | 0.068149 | 0.041053 | 0.095264 | 8.32E-07 | Logistic-binary | Operation: Hip replacement | Verbal interview | Operations | 0.001969 | 0.017717 |
| 1518 | 331476 | -0.01784 | -0.025 | -0.01068 | 1.04E-06 | Ordered-logistic | Hot drink temperature | Touchscreen | Lifestyle and environment | 0.002219 | 0.022185 |
| 20004#1455 | 321660/13267(334927) | 0.043287 | 0.025731 | 0.060851 | 1.35E-06 | Logistic-binary | Operation: Gallbladder removal | Verbal interview | Operations | 0.002622 | 0.028841 |
| 20153 | 105066 | 0.006068 | 0.003505 | 0.008631 | 3.48E-06 | Linear | Forced expiratory volume in 1-second (FEV1), predicted | Physical measures | Spirometry | 0.006168 | 0.074015 |
| 23128 | 328871 | 0.007905 | 0.00451 | 0.0113 | 5.03E-06 | Linear | Trunk fat mass | Physical measures | Anthropometry | 0.007729 | 0.107093 |
| 23127 | 328890 | 0.007144 | 0.004074 | 0.010214 | 5.08E-06 | Linear | Trunk fat percentage | Physical measures | Anthropometry | 0.007729 | 0.108204 |
| 41200#W381 | 331620/3348(334968) | 0.076387 | 0.042177 | 0.110624 | 1.22E-05 | Logistic-binary | Procedure: Hip joint replacement | Hospital in-patient | Operations | 0.017173 | 0.25916 |
| 23106 | 329066 | 0.005758 | 0.003171 | 0.008346 | 1.29E-05 | Linear | Impedance of whole body | Physical measures | Anthropometry | 0.017173 | 0.274773 |
| 4101 | 105662 | -0.01262 | -0.01834 | -0.0069 | 1.55E-05 | Linear | Heel broadband ultrasound attenuation (left) | Physical measures | Bone-densitometry of heel | 0.019386 | 0.329558 |
| 41200#L671 | 334548/420(334968) | -0.20693 | -0.3009 | -0.11234 | 1.7E-05 | Logistic-binary | Procedure: Biopsy of artery NEC | Hospital in-patient | Operations | 0.02013 | 0.362334 |
| 2139 | 295123 | 0.007608 | 0.004115 | 0.011101 | 1.96E-05 | Linear | Age first had sexual intercourse | Touchscreen | Lifestyle and environment | 0.021999 | 0.417972 |
| 41221#7 | 6272/1025(7297) | 0.143868 | 0.076732 | 0.211277 | 2.76E-05 | Logistic-binary | Delivery methods | Hospital in-patient | Maternity | 0.027605 | 0.58879 |
| 4123 | 105639 | -0.01243 | -0.01825 | -0.0066 | 2.87E-05 | Linear | Heel quantitative ultrasound index (QUI), direct entry (right) | Physical measures | Bone-densitometry of heel | 0.027605 | 0.611416 |
| 4125 | 105639 | -0.01242 | -0.01825 | -0.0066 | 2.88E-05 | Linear | Heel bone mineral density (BMD) T-score, automated (right) | Physical measures | Bone-densitometry of heel | 0.027605 | 0.612793 |
| 41204#D376 | 334945/23(334968) | -0.76439 | -1.10866 | -0.39227 | 2.98E-05 | Logistic-binary | Diagnoses - secondary ICD10 | Hospital in-patient | Diagnoses | 0.027605 | 0.634911 |
| 4124 | 105582 | -0.01219 | -0.01801 | -0.00637 | 4.06E-05 | Linear | Heel bone mineral density (BMD) (right) | Physical measures | Bone-densitometry of heel | 0.035992 | 0.86381 |
| 2375 | 149748 | 0.02645 | 0.013701 | 0.039198 | 4.78E-05 | Ordered-logistic | Relative age of first facial hair | Touchscreen | Sex-specific factors | 0.040688 | 1 |
| 20015 | 333928 | 0.005291 | 0.00273 | 0.007851 | 5.13E-05 | Linear | Sitting height | Physical measures | Anthropometry | 0.042047 | 1 |
| 1528 | 308989 | -0.0136 | -0.02021 | -0.00699 | 5.55E-05 | Ordered-logistic | Water intake | Touchscreen | Lifestyle and environment | 0.042275 | 1 |
| 20004#1458 | 295440/39487(334927) | -0.02165 | -0.03218 | -0.01112 | 5.56E-05 | Logistic-binary | Operation code | Verbal interview | Operations | 0.042275 | 1 |
| 4106 | 105665 | -0.01194 | -0.01779 | -0.0061 | 6.12E-05 | Linear | Heel bone mineral density (BMD) T-score, automated (left) | Physical measures | Bone-densitometry of heel | 0.043507 | 1 |
| 4104 | 105665 | -0.01194 | -0.01778 | -0.0061 | 6.13E-05 | Linear | Heel quantitative ultrasound index (QUI), direct entry (left) | Physical measures | Bone-densitometry of heel | 0.043507 | 1 |

Table S6 Descriptions of the continuous phenotypes followed-up from the MR-pheWAS.

|  | **Impedance (left arm) [ohms]** | **Impedance (right arm) [ohms]** | **Pulse (automated) [bpm]** | **Standing Height [cm]** | **Impedance (whole body) [ohms]** | **Heel broadband ultrasound attenuation (right) [dB/MHz] †** |
| --- | --- | --- | --- | --- | --- | --- |
| **N** | 329070 | 329059 | 312949 | 334232 | 329066 | 105633 |
| **N females** | 177227 | 177217 | 168205 | 179844 | 177217 | 56736 |
| **N males** | 151843 | 151842 | 144744 | 154388 | 151849 | 48897 |
| **Minimum** | 58 | 56 | 30 | 75 | 51 | 0 |
| **Median** | 328 | 321 | 68 | 168 | 594 | 77.02 |
| **Mean** | 331.5 | 324.7 | 69.5 | 168.8 | 598.99 | 78.5 |
| **Maximum** | 999 | 998 | 174 | 209 | 998 | 209.6 |
| **NA's** | 5898 | 5909 | 22019 | 736 | 5902 | 229335 |

* For the water intake phenotype values were removed if they were >40 glasses water/day as they were deemed implausible.

† Bone densitometry of heel: heel broadband ultrasound attenuation performed on only a subset of the full UK Biobank cohort.

Table S7 Descriptions of binary phenotypes followed-up from the MR-pheWAS.

|  | **Gallbladder removal (cholecystectomy)** | | **Hip replacement/revision** | | **High cholesterol report** | | **Cholesterol medication** | |
| --- | --- | --- | --- | --- | --- | --- | --- | --- |
| **Category** | **N (female/male)** | **Percent (female/male)** | **N (female/male)** | **Percent (female/male)** | **N (female/male)** | **Percent (female/male)** | **N (female/male)** | **Percent (female/male)** |
| **1 (controls)** | 321701 (169863/151838) | 96.0393 (94.2552/98.117) | 329567 (177239/152328) | 98.3876 (98.3481/98.4336) | 294108 (162855/131253) | 87.8018 (90.3666/84.8151) | 119409 (0/119409) | 77.1615 (0/77.1615) |
| **2 (cases)** | 13267 (10353/2914) | 3.9607 (5.7448/1.883) | 5401 (2977/2424) | 1.6124 (1.6519/1.5664) | 40860 (17361/23499) | 12.1982 (9.6334/15.1849) | 35343 (0/35343) | 22.8385 (0/22.8385) |

Table S8 Descriptions of categorical phenotypes followed-up from the MR-pheWAS

|  | **Alcohol frequency** | | **Hot drink temperature preference** | |
| --- | --- | --- | --- | --- |
| **Category** | **N (females/males) *** | **Percent (females/males) *** | **N (females/males) †** | **Percent (females/males) †** |
| 1 | 71854 (30761/41093) | 21.451 (17.069/26.5541) | 56076 (33456/22620) | 16.7407 (18.5644/14.6169) |
| 2 | 81049 (39077/41972) | 24.196 (21.6834/27.1221) | 224953 (118024/106929) | 67.1566 (65.4903/69.097) |
| 3 | 88015 (47558/40457) | 26.2756 (26.3894/26.1431) | 50447 (26824/23623) | 15.0602 (14.8844/15.2651) |
| 4 | 37052 (23568/13484) | 11.0614 (13.0776/8.7133) | - | - |
| 5 | 35029 (24941/10088) | 10.4574 (13.8395/6.5188) | - | - |
| 6 | 21736 (14186/7550) | 6.489 (7.8717/4.8788) | - | - |

*Alcohol frequency categories are 1=”Daily or almost daily”, 2=”Three of four times a week”, 3=”Once or twice a week”, 4=”One to three times a month”, 5=”Special occasions only”, 6=“Never”

†Hot drink temperature categories are 1=”Very hot”, 2=”Hot”, 3=”Warm”

Table S9 Results of MR-PRESSO sensitivity analysis.

| **MR analysis** | **Causal Estimate** | **Sd** | **T-stat** | **P-value** | **Outcome** |
| --- | --- | --- | --- | --- | --- |
| Raw | 5.362975 | 3.518067 | 1.52441 | 0.161744 | Impedance right arm |
| Outlier-corrected | 7.232182 | 0.424767 | 17.02625 | 0.000441 | Impedance right arm |
| Raw | 5.459744 | 3.57617 | 1.526701 | 0.16118 | Impedance left arm |
| Outlier-corrected | 7.215749 | 0.451911 | 15.96721 | 0.000534 | Impedance left arm |
| Raw | -1.45749 | 0.734381 | -1.98464 | 0.078471 | Pulse rate |
| Outlier-corrected | -1.23387 | 0.28219 | -4.3725 | 0.003264 | Pulse rate |
| Raw | 5.639839 | 6.003491 | 0.939427 | 0.372038 | Impedance whole body |
| Outlier-corrected | 8.869079 | 1.391626 | 6.373175 | 0.00782 | Impedance whole body |
| Raw | 0.860482 | 0.512828 | 1.677917 | 0.127674 | Standing height |
| Outlier-corrected | 1.639613 | 0.176921 | 9.267477 | 0.002659 | Standing height |
| Raw | -0.19288 | 0.151154 | -1.27604 | 0.233886 | Alcohol frequency |
| Outlier-corrected | -0.11413 | 0.05438 | -2.09869 | 0.074014 | Alcohol frequency |
| Raw | -2.81379 | 0.676649 | -4.15841 | 0.002453 | Heel broadband ultrasound attenuation (right) |
| Outlier-corrected | - | - | - | - | Heel broadband ultrasound attenuation (right) |
| Raw | 0.728653 | 0.128516 | 5.669748 | 0.000306 | Hip replacement/revision |
| Outlier-corrected | - | - | - | - | Hip replacement/revision |
| Raw | 0.488908 | 0.261524 | 1.869458 | 0.09438 | Gallbladder removal |
| Outlier-corrected | 0.235612 | 0.121493 | 1.939312 | 0.100538 | Gallbladder removal |
| Raw | -0.36608 | 0.153441 | -2.38582 | 0.040836 | Cholesterol medication |
| Outlier-corrected | -0.2701 | 0.075287 | -3.58763 | 0.007109 | Cholesterol medication |
| Raw | -0.29648 | 0.217062 | -1.36587 | 0.205136 | High cholesterol report |
| Outlier-corrected | -0.15396 | 0.082336 | -1.86992 | 0.098419 | High cholesterol report |
| Raw | -0.19346 | 0.07803 | -2.47926 | 0.035034 | Hot drink temperature preference |
| Outlier-corrected | -0.17106 | 0.027805 | -6.15222 | 0.000467 | Hot drink temperature preference |

Table S10 Results of the replication two-sample MR analysis for heart rate using publically available GWAS summary results in MR-Base.

| **Analysis** | **Outcome** | **Method** | **N SNP** | **b*** | **SE** | **P-value** | **lower CI** | **upper CI** |
| --- | --- | --- | --- | --- | --- | --- | --- | --- |
| SHBG score | Heart rate† | Weighted median | 10 | -0.414 | 0.493 | 0.400 | -1.381 | 0.552 |
| SHBG score | Heart rate | Inverse variance weighted (multiplicative random effects) | 10 | -0.610 | 0.474 | 0.198 | -1.539 | 0.318 |
| SHBG score | Heart rate | Inverse variance weighted (fixed effects) | 10 | -0.610 | 0.357 | 0.087 | -1.310 | 0.090 |
| SHBG score | Heart rate | MR Egger | 10 | -0.448 | 0.891 | 0.629 | -2.195 | 1.299 |
| SHBG score | Heart rate | MR Egger (bootstrap) | 10 | 0.014 | 0.913 | 0.479 | -1.776 | 1.804 |
| SHBG score sensitivity | Heart rate | Weighted median | 9 | -0.383 | 0.484 | 0.428 | -1.331 | 0.565 |
| SHBG score sensitivity | Heart rate | Inverse variance weighted (multiplicative random effects) | 9 | -0.383 | 0.364 | 0.293 | -1.096 | 0.331 |
| SHBG score sensitivity | Heart rate | Inverse variance weighted (fixed effects) | 9 | -0.383 | 0.366 | 0.296 | -1.100 | 0.335 |
| SHBG score sensitivity | Heart rate | MR Egger | 9 | -0.814 | 0.660 | 0.257 | -2.107 | 0.478 |
| SHBG score sensitivity | Heart rate | MR Egger (bootstrap) | 9 | 0.123 | 0.853 | 0.445 | -1.549 | 1.796 |

†where outcome dataset is summary results from den Hoed et al. (2013) study of heart rate, measured in beats per minute (bpm)

*change in heart rate (bpm) per unit increase in log SHBG

# References

1. Millard LAC, Davies NM, Gaunt TR, Davey Smith G, Tilling K. Software Application Profile: PHESANT: a tool for performing automated phenome scans in UK Biobank. Int J Epidemiol. 2017.

2. Shim H, Chasman DI, Smith JD, Mora S, Ridker PM, Nickerson DA, et al. A Multivariate Genome-Wide Association Analysis of 10 LDL Subfractions, and Their Response to Statin Treatment, in 1868 Caucasians. PLOS ONE. 2015;10(4):e0120758.

3. Coviello AD, Haring R, Wellons M, Vaidya D, Lehtimäki T, Keildson S, et al. A genome-wide association meta-analysis of circulating sex hormone-binding globulin reveals multiple Loci implicated in sex steroid hormone regulation. PLoS genetics. 2012;8(7):e1002805.
